# Chromosome-Level Genome Assembly of *Solanum carolinense*

**DOI:** 10.3390/plants15172681

**Published:** 2026-08-31

**Authors:** Luyue Shan, Xiaoling Song, Jianguo Fu, Weimin Dai, Jing Wu, Jinggan Li, Neng Wan, Jianguo Liang, Yuanwei Ma

**Affiliations:** 1 Weed Research Laboratory, College of Life Sciences, Nanjing Agricultural University, Nanjing 210095, China; shily@stu.njau.edu.cn (L.S.); daiweimin4@njau.edu.cn (W.D.); 2 Nanjing Customs Animal, Plant and Food Inspection Center, Nanjing 210019, China; 13851670320@163.com (J.W.); lijgcaci201622@163.com (J.L.); 3 Department of Mathematics, Faculty of Science, The Chinese University of Hong Kong, Shatin, N.T., Hong Kong SAR 999077, China; nengwan@math.cuhk.edu.hk; 4 Nanjing iFlashDx Biotechnology Limited, Nanjing 211100, China; jayliang@iflashdx.com (J.L.); marioma@iflashdx.com (Y.M.)

**Keywords:** *Solanum carolinense*, genome sequencing, chromosome assembly

## Abstract

Horsenettle (*Solanum carolinense* L.) is a noxious weed widely distributed across North America and increasingly invasive in other regions. Its strong environmental adaptability, complex defense strategies, and distinctive reproductive traits make it an important model for studying plant–herbivore coevolution. However, the absence of high-quality genomic resources has limited deeper investigation into its adaptive evolutionary mechanisms. In this study, we generated a chromosome-level reference genome assembly for *S. carolinense* using an integrated approach combining PacBio HiFi long-read sequencing, Illumina second-generation sequencing, and Hi-C chromatin interaction scaffolding. The final genome assembly had a total length of 915.40 Mb, with a contig N50 of 51.06 Mb and a scaffold N50 of 73.17 Mb; 96.05% of the sequences were successfully anchored onto 12 pseudochromosomes. The genome was characterized by a high proportion of repetitive sequences (73.64%) and substantial heterozygosity (1.13%), consistent with a highly repetitive and moderately high heterozygous genome. BUSCO analysis indicated that the chromosome-level genome assembly of *S. carolinense* reached a completeness score of 94.8%. A total of 32,206 protein-coding genes were annotated, of which 97.95% received functional annotations. The evaluation of the annotated protein-coding gene set returned a completeness value of 94.9%. This reference genome provides a valuable resource for advancing research on the adaptive evolution of weedy Solanaceae species, supports the development of more effective management strategies for this troublesome species, and offers a technical reference for assembling other highly heterozygous weed genomes.

## 1. Introduction

*Solanum carolinense* is a perennial herbaceous species in the Solanaceae family, native to the southeastern United States and widely recognized as a noxious weed [1]. The species has spread throughout the United States, Canada, and at least 36 countries and regions across Oceania, Europe, and Asia [2]. In China, it was first discovered in Zhejiang Province, China, in 2006, with subsequent distribution records reported on offshore islands of Taizhou and Wenzhou cities [3,4]. *S. carolinense* is a troublesome weed that infests major food crops such as corn, potato, and peanut, as well as vegetables and forage crops [5]. In addition, *S. carolinense* serves as a host for key pests, including the Colorado potato beetle and carmine spider mite, and for several Solanaceae-infecting viruses, thereby increasing pest and disease pressure in cropping systems [6]. In 2007, it was included in China’s Catalog of Imported Plant Quarantine Pests [7].

Beyond its agricultural impact, *S. carolinense* is also an important model for studying plant–herbivore interactions [8]. It exhibits distinctive genetic traits, including a plastic self-incompatibility system [9], diverse stress resistance and defense mechanisms, and strong asexual reproductive capacity [7]. Its defense strategies include both constitutive and inducible mechanisms: physical defenses such as non-glandular trichomes and prickles [10], and chemical defenses including polyphenols, glycoalkaloids, and trypsin inhibitors [11,12]. Its plastic self-incompatibility system [13], andromonoecious floral sex expression [14], and tolerance to inbreeding depression may provide flexibility in reproductive strategies. Furthermore, *S. carolinense* can thrive in sandy and gravelly substrates [15] and propagate via seeds as well as root fragments, rendering its control extremely difficult [5]. Although this invasive weed poses severe threats to global food security, existing control measures remain limited, with management largely dependent on chemical herbicides that may adversely affect crop fertility. These include autumn-only picloram application to inhibit root proliferation, as well as tank mixtures of picloram and glyphosate for mature fruiting individuals [5]. With many regions of China now suitable for its establishment [3,4], thorough studies on its genetic differentiation, allelopathy, and dispersal capacity are urgently needed.

Genome sequencing has become a core tool for elucidating plant genetic architecture, uncovering molecular mechanisms underlying key traits, and supporting crop improvement and sustainable agriculture [16]. In the genus *Solanum*, chloroplast genomes have been particularly informative. The chloroplast genome of *Solanum elaeagnifolium* Cav. is 155,049 bp [17]. Species-specific SSR markers developed for *S. elaeagnifolium* provide valuable tools for assessing genetic diversity and tracing population origins in this species and its relatives [18]. Similarly, the chloroplast genome of *Solanum dulcamara* L. (155,580 bp) [19] exhibits a typical angiosperm quadripartite structure and low genetic polymorphism, with only weak geographical and ecological structuring among populations [20]. While chloroplast genomes offer useful insights, whole-genome sequencing provides the complete nuclear sequence information required to investigate species evolution, gene function, regulatory networks, and adaptive mechanisms [21,22].

Among *Solanum* weed species to date, only *Solanum rostratum* Dunal and *Solanum torvum* Sw. have undergone high-quality chromosome-level genome assembly. The final assembled genome of *S. rostratum* spans 869.69 Mb, with a BUSCO completeness score of 99.5% for pseudochromosomes [23]. The assembled genome of *S. torvum* is approximately 1.25 Gb in size, achieving a BUSCO completeness of 98% [24]. Previously, only the chloroplast genome of *S. carolinense* has been assembled and characterized [25] while a high-quality nuclear chromosome-level assembly remains absent. A high-quality chromosome-level nuclear genome would enable systematic exploration of the genetic basis of invasive adaptability, reproductive biology, herbicide target sites, non-target herbicide-resistance loci, and structural variants that may facilitate rapid evolution. Such resources are essential for deciphering the molecular mechanisms driving their invasiveness and for developing evidence-based management strategies.

This study employed a multi-platform sequencing approach to generate a high-quality chromosome-level reference genome for *S. carolinense.* We integrated Pacific Biosciences (PacBio) High-Fidelity (HiFi) long-read sequencing (characterized by long read lengths and superior base accuracy), Illumina next-generation short-read sequencing (notable for high throughput and cost-effectiveness) [26], and high-throughput, high-resolution chromosome conformation capture (Hi-C), a technology enabling chromosome-scale scaffolding and the integration of three-dimensional chromatin architecture with multi-omics datasets [27]. The final assembly was comprehensively annotated for protein-coding genes, non-coding RNAs, and repetitive elements. We also identified candidate genes associated with defense responses and reproduction. These genomic resources will establish a robust molecular foundation for future research on adaptive evolution, defense regulation, reproductive system evolution, and invasive dispersal mechanisms in *S. carolinense*.

## 2. Results

### 2.1. Sequencing Data Processing

The sequencing data for the *S. carolinense* genome were generated using PacBio HiFi long reads, next-generation sequencing (NGS) short reads, and Hi-C reads. Following quality control and filtering, a total of 99.89 Gb of HiFi clean data, 396.96 Gb of NGS clean data, and 88.40 Gb of Hi-C clean data were obtained (Table 1).

### 2.2. Preliminary Genome Assembly Using HiFi Sequencing Data

A total of 99.89 Gb of high-quality HiFi reads were generated, with an average read length of 18,660.8 bp and an N50 of 18,384 bp. Genome assembly using hifiasm [28] produced a draft genome of 915.39 Mb, consisting of 128 contigs with a contig N50 of 51.06 Mb (Table 2). To assess assembly accuracy, the NGS short reads were remapped, resulting in an alignment rate of 99.90% and a coverage of 98.45%. These metrics indicated excellent consistency between the assembled genome and the raw sequencing data.

### 2.3. Genome Characterization

**Figure 1:**
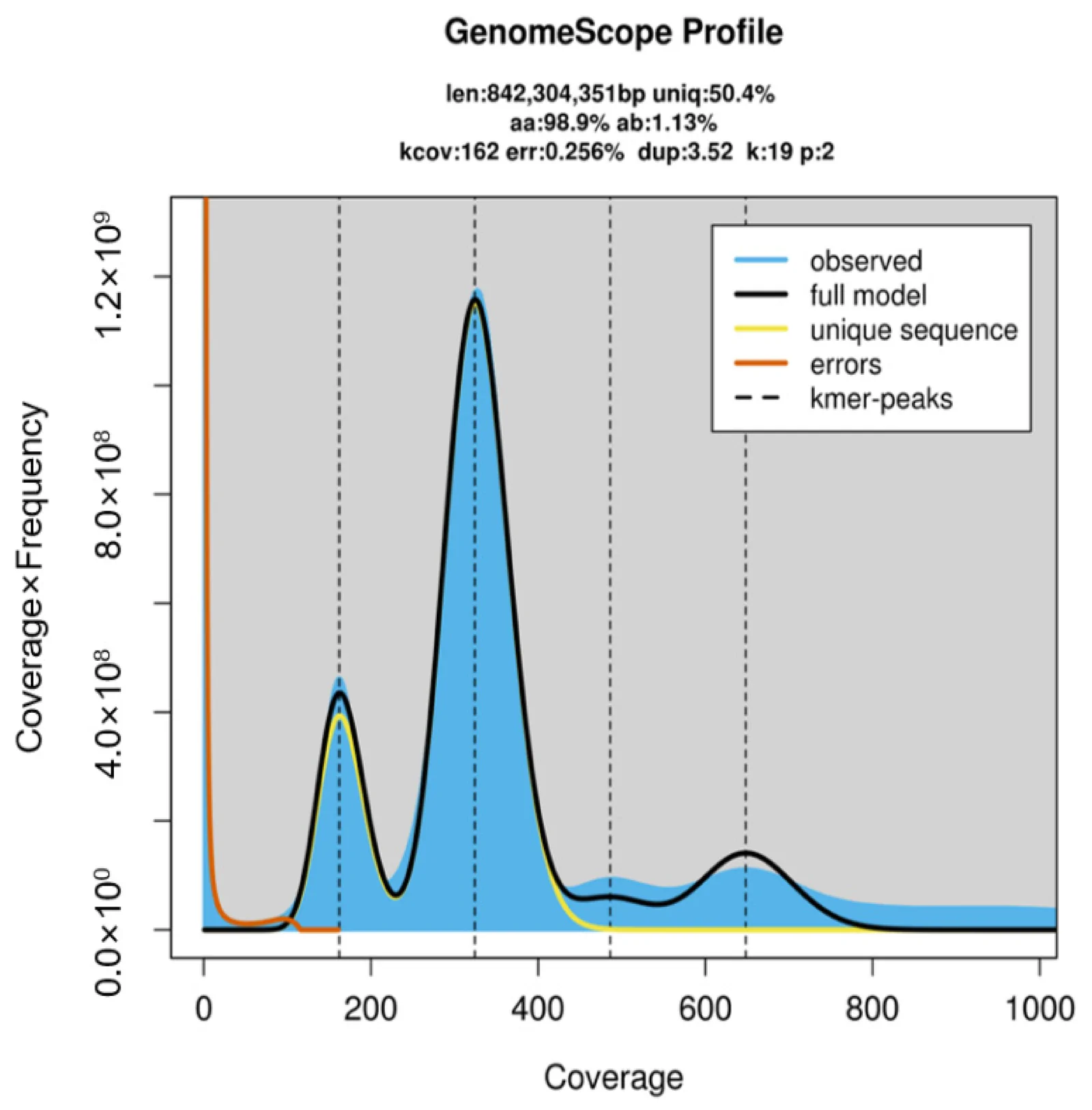
K-mer spectrum analysis of *S. carolinense*. The x-axis represents the sequencing coverage depth, and the y-axis denotes the product of coverage and k-mer frequency. The blue area indicates the observed k-mer distribution, while the black line represents the fitted full model generated by GenomeScope. The yellow and orange curves correspond to unique genomic sequences and sequencing errors, respectively. Vertical dashed lines mark the primary k-mer peaks for homozygous and heterozygous regions.

To preliminarily characterize the genome, k-mer analysis was performed based on the NGS data. This analysis estimated a genome size of roughly 842 Mb, alongside a heterozygosity rate of 1.13% and a repeat content of 49.64%. The k-mer spectrum exhibited two peaks, where the peak at half-coverage depth indicated heterozygous loci and the main peak at full-coverage depth represented homozygous regions. These results indicated that the *S. carolinense* genome is highly repetitive, with moderate-to-high heterozygosity. The k-mer distribution is presented in Figure 1.

### 2.4. Hi-C-Assisted Chromosome-Level Scaffolding and Quality Evaluation

**Figure 2:**
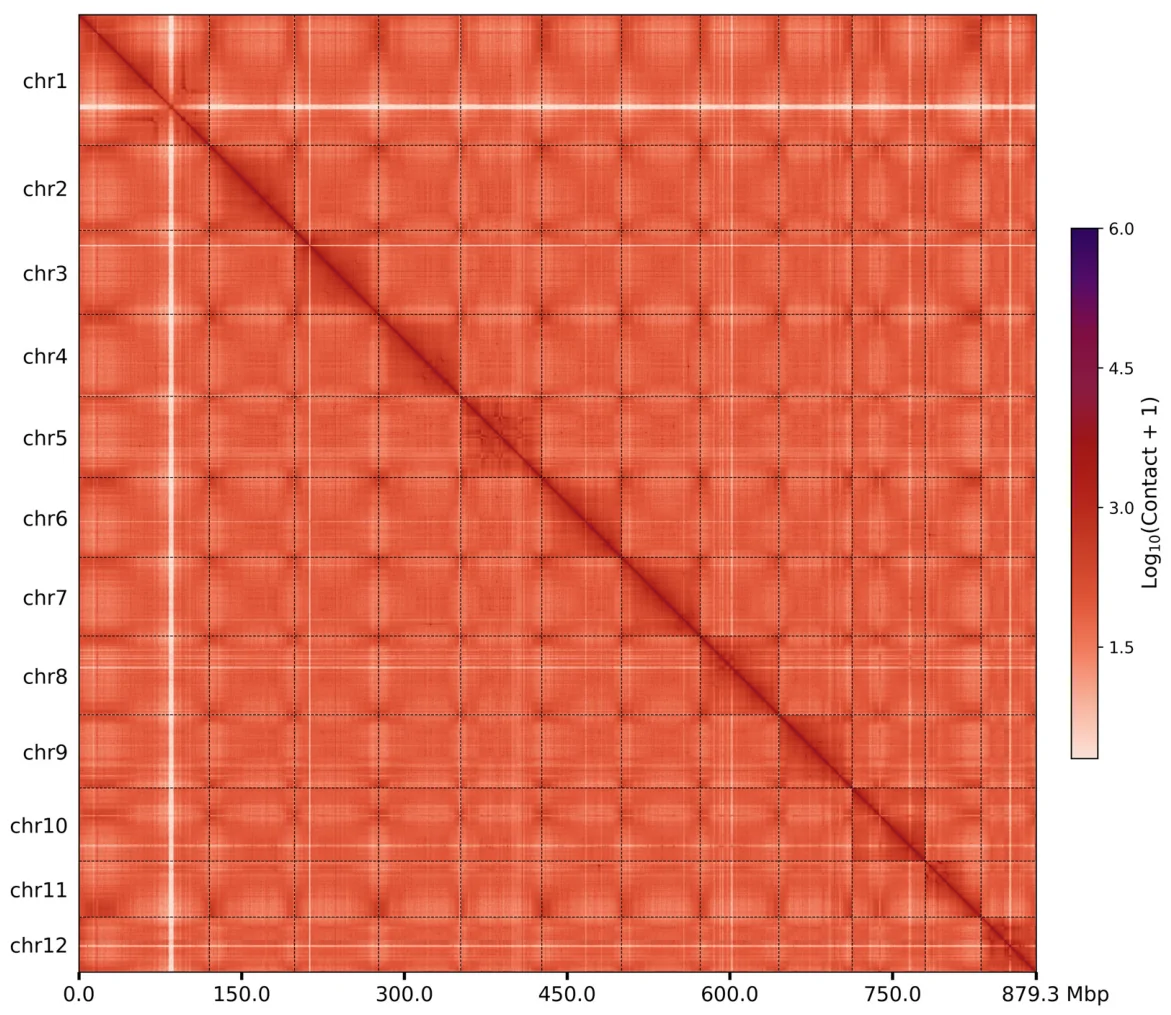
Hi-C chromatin contacts heatmap of the *S. carolinense* genome assembly. The x and y axes denote genomic coordinates, and the color depth reflects the strength of chromatin interactions. Clear separation of 12 pseudochromosomes is observed; strong diagonal signals and weak off-diagonal signals conform to Hi-C interaction rules, confirming effective chromosome anchoring.

The final chromosome-level genome assembly of *S. carolinense* was 915.40 Mb in length, with a GC content of 35.61% and a scaffold N50 of 73.17 Mb (Table 2), indicating high continuity and completeness of the assembly that met the requirements for subsequent gene annotation and functional analyses. Following Hi-C scaffolding and manual curation, 879.26 Mb of contig sequences (96.05% of the assembled genome) were anchored to 12 pseudochromosomes. This anchoring ratio aligned with the value described above, while the remaining approximately 3.95% of the assembled sequences corresponded to unplaced chrUn contigs. The chromosome-level assembly of the *S. carolinense* genome is visualized as a normalized Hi-C chromatin contact heatmap in Figure 2. The contact heatmap shows strong intra-chromosomal interaction signals along the diagonal and weak inter-chromosomal interactions, confirming the correct anchoring of scaffolds to individual chromosomes with minimal misjoins or chimeric assemblies.

**Figure 3:**
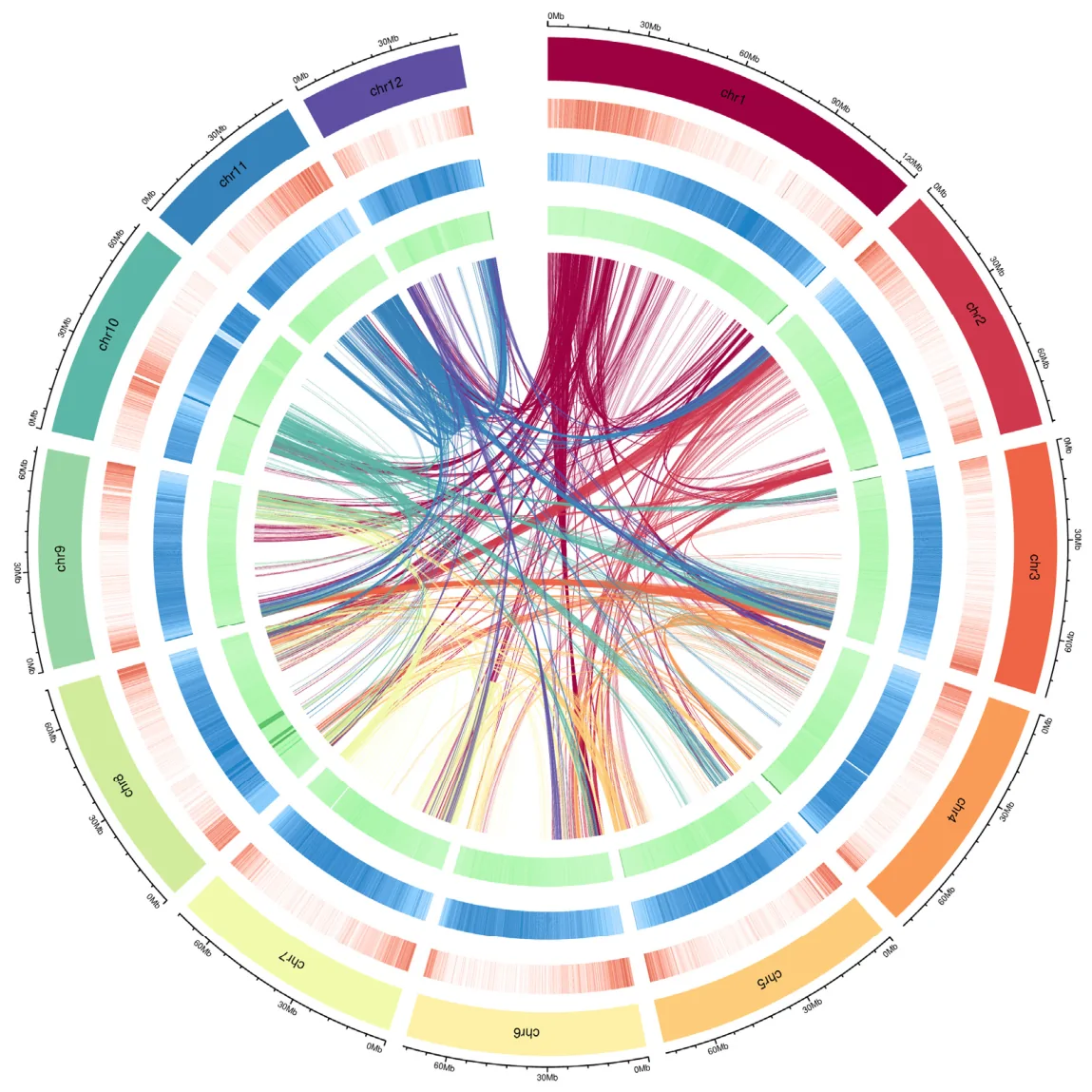
Genomic characteristics of 12 pseudochromosomes of *S. carolinense*. This Circos diagram displays chromosome length, gene density, repetitive sequence density, GC content and collinearity blocks. The outer three rings show gene density, repeat density and GC content calculated in 10,000 bp sliding windows, and central multicolored links represent collinearity blocks among pseudochromosomes.

Genome completeness was evaluated with Benchmarking Universal Single-Copy Orthologs (BUSCO, v5.7.0) [29] in genome mode against the embryophyta_odb10 database, with the full resulting assembly (915.40 Mb), yielding a completeness of 94.8% (Table 3), indicating excellent completeness of the assembly. A Circos diagram integrating chromosome length, gene density, repeat sequence density, GC content, and collinearity blocks was constructed to provide an overview of the genomic characteristics (Figure 3).

### 2.5. Genome Annotation and Quality Evaluation

#### 2.5.1. Structural Annotation

Transposable elements (TEs) of *S. carolinense* were annotated via de novo prediction and Repbase homology searching (Table 4). In total, 674.12 Mb of repetitive sequences were identified, accounting for 73.64% of the genome. LTR retrotransposons represented the largest fraction of repetitive elements, occupying 54.16% of the assembled genome. Within the LTR superfamily, the abundance of Gypsy-type elements (25.41%) was substantially higher than that of Copia-type elements (5.14%). DNA transposons and LINEs constituted the secondary major repeat categories, making up 10.98% and 7.64% of the genome, respectively. SINE elements occupied a small fraction (0.88%) of the assembly. In addition, unknown repetitive sequences accounted for 4.26% of the genome.

Repeat-sequence annotation was performed by combining public-database alignment and de novo annotation. Multiple types of non-coding RNAs (ncRNAs) were identified in the genome, and their statistics are summarized in Table 5. Among these ncRNAs, ribosomal RNA (rRNA) was the most abundant class, with a total length of 1,931,473 bp, occupying 0.211% of the genome. Within rRNA, the 5S rRNA exhibited the highest copy number (14,984), followed by 18S, 28S and 5.8S rRNA. MicroRNA (miRNA), transfer RNA (tRNA), and small nuclear RNA (snRNA) showed relatively low genomic proportions, accounting for 0.008%, 0.0095%, and 0.0103% of the genome, respectively. The snRNA category was further divided into CD-box, HACA-box and splicing-related snRNAs. No small Cajal body-specific RNA (scaRNA) sequences were detected in the present genome assembly.

A total of 32,206 protein-coding genes were predicted and annotated in the *S. carolinense* genome. Gene prediction was performed using multiple complementary strategies, including ab initio prediction (GlimmerHMM, AUGUSTUS), homology-based search (Miniprot, with three related *Solanum* species as references), and RNA-seq-evidence-based prediction (TransDecoder). All predicted gene evidence was then integrated using the MAKER pipeline to generate the consensus gene set. The final gene set had an average gene length of 5209 bp, with a coding sequence (CDS) length of 1149 bp, 4.78 exons per gene, an average exon length of 312 bp, and an average intron length of 982 bp (Table 6).

#### 2.5.2. Quality Evaluation

BUSCO (v5.7.0) [29] evaluation based on the embryophyta_odb10 dataset was performed to assess the completeness of both the genome assembly and gene annotation (Table 3). For the genome assembly, 94.8% of the conserved orthologs were identified as complete, with only 0.4% fragmented and 4.8% missing orthologs. Similarly, the annotated protein set exhibited a comparable outcome, reaching 94.9% complete BUSCOs, accompanied by 0.5% fragmented and 4.6% missing. The high proportion of complete BUSCOs and the low fractions of fragmented and missing genes collectively demonstrated the high continuity and integrity of the assembled genome, as well as the high accuracy of the predicted gene models.

#### 2.5.3. Functional Annotation

Multi-database functional annotation was conducted for the 32,206 predicted protein-coding genes (Table 7). Of these, 31,547 genes were functionally annotated against at least one database, with an annotation rate of 97.95%. Gene Ontology (GO) [30] terms and Kyoto Encyclopedia of Genes and Genomes (KEGG) with KofamKOALA [31] pathway information were assigned to 57.60% and 32.03% of genes, respectively, while identifiable eukaryotic Orthologous Groups (KOG) classifications were retrieved for merely 10.94% of genes. Non-Redundant protein database (NR) [32], UniProt [33], and InterPro [34] databases yielded annotation rates exceeding 94%, whereas Pfam (Protein Families Database) [35] annotations covered 70.58% of all genes.

**Figure 4:**
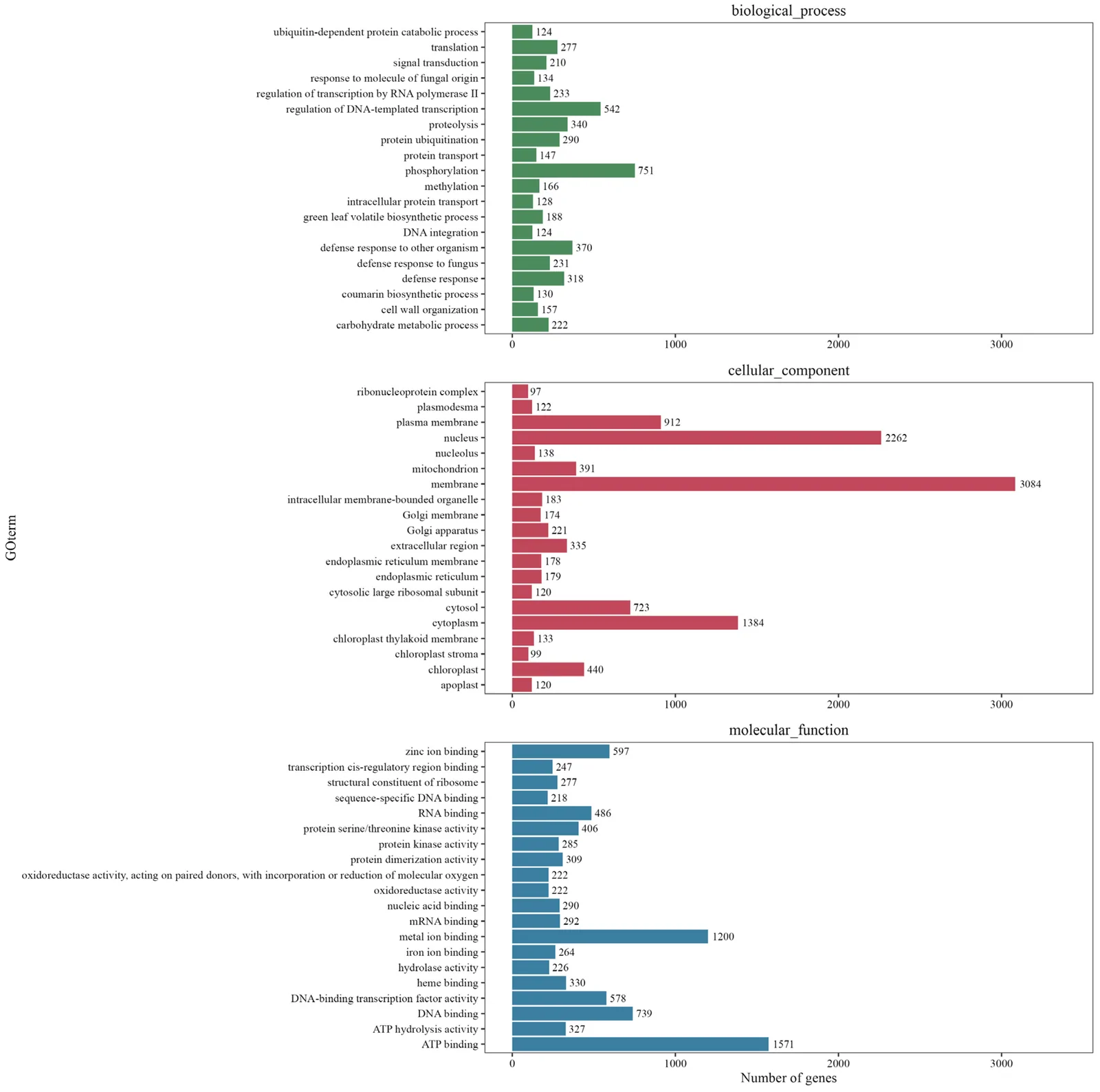
GO functional enrichment analysis of annotated genes in *S. carolinense*. The top 20 significantly enriched GO terms categorized into Biological Process (BP), Cellular Component (CC), and Molecular Function (MF) were sorted by significance level (*p*-value) and visualized using bar charts. The horizontal axis represents the number of annotated genes assigned to each GO term, while GO functional items are arranged on the vertical axis. GO entries within each subcategory are ranked in descending order based on the total number of matched genes.

**Figure 5:**
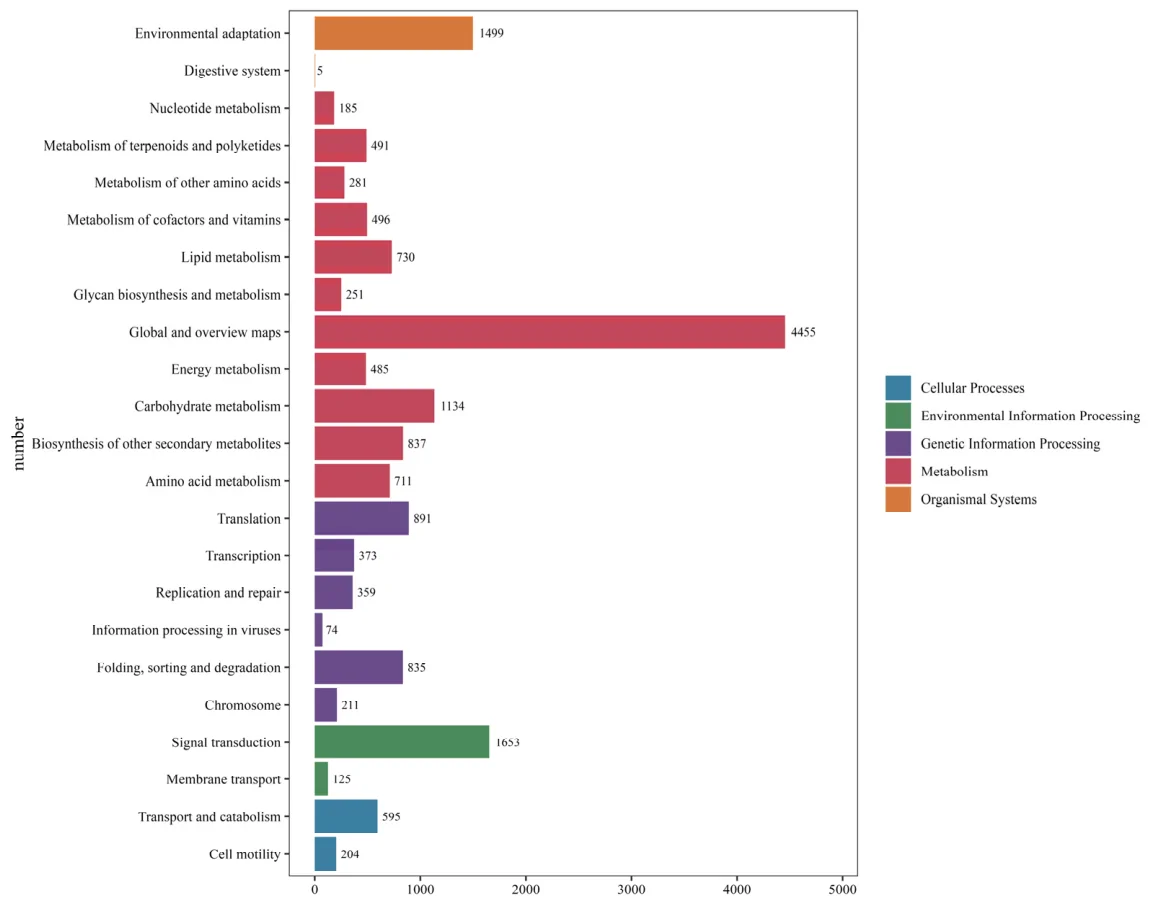
KEGG pathway enrichment analysis of predicted protein-coding genes in *S. carolinense*. The significantly enriched KEGG pathways were divided into five major functional categories and visualized as horizontal bars. The x-axis indicates the number of matched genes for each secondary KEGG pathway, and the y-axis lists pathway items. The color of each bar corresponds to its top-level KEGG functional category as defined in the legend.

**Figure 6:**
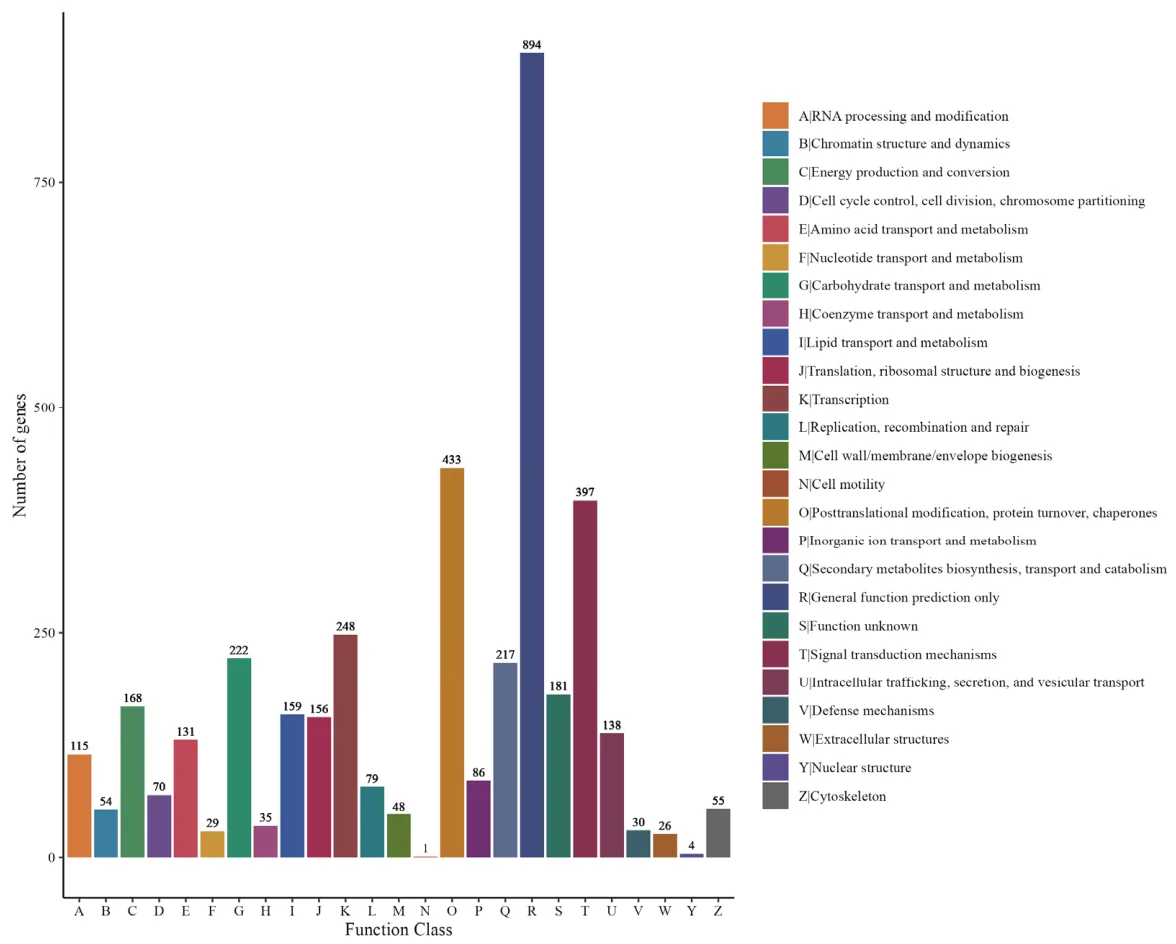
KOG functional classification analysis of predicted protein-coding genes in *S. carolinense*. Annotated genes were classified into 25 alphabetically coded KOG functional classes and displayed as vertical bars. The y-axis indicates the gene count of each functional class, and the x-axis shows the class codes. Bar colors correspond to distinct functional groups as illustrated in the legend.

**Figure 7:**
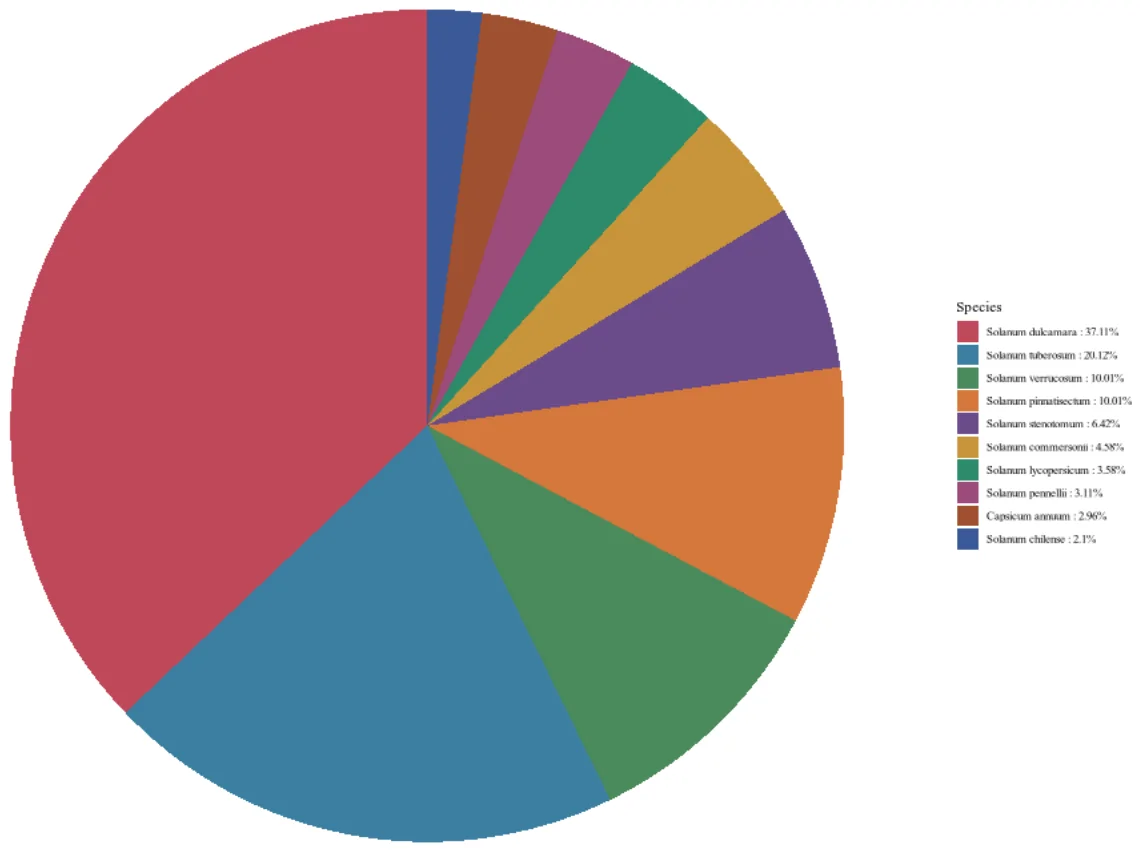
NR database homology annotation results of predicted protein-coding genes in *S. carolinense*. The pie chart shows the percentage of genes with the best match to each solanaceous reference species, and each colored slice corresponds to one species as annotated in the legend.

GO enrichment analysis (Figure 4) revealed that the annotated genes are involved in a wide range of biological processes, including phosphorylation, regulation of DNA-templated transcription, and defense responses to other organisms. Subcellular localization analysis showed that these proteins were predominantly distributed across the cell membrane, nucleus, and cytoplasm. Molecular functions primarily included ATP binding, metal ion binding, and DNA binding. KEGG pathway enrichment analysis (Figure 5) revealed that the candidate genes were primarily enriched in global and overview metabolism, signal transduction, and environmental adaptation pathways. KOG functional classification (Figure 6) revealed that the largest proportion of annotated genes fell into categories of general function prediction only, post-translational modification, protein turnover, chaperones, and signal transduction mechanisms. Consistent enrichment of genes associated with post-translational modification and molecular chaperone systems highlighted the central role of protein homeostasis in *S. carolinense*. NR homology annotation (Figure 7) revealed that *S. dulcamara* (37.11%), *Solanum tuberosum* L. (20.12%) and *Solanum verrucosum* Schltdl. (10.01%) exhibited the highest sequence similarity to *S. carolinense* genes. All of the top 10 matched species, except *Capsicum annuum* L., were members of the genus *Solanum*, which demonstrated the tight phylogenetic affinity of *S. carolinense* within Solanaceae.

## 3. Discussion

In this study, we integrated PacBio HiFi long-read, Illumina short-read, and Hi-C chromatin interaction sequencing data to perform chromosome-level genome assembly and systematic quality assessment of *S. carolinense*. Sequencing data generated from multiple platforms exhibited sufficient coverage and high quality, and the complementary advantages of these sequencing technologies laid a solid foundation for high-quality genome assembly [21]. HiFi long reads ensured contig-level contiguity, Illumina short reads enabled accurate base-level validation and genomic feature characterization, and Hi-C data provided valid chromatin interaction information for chromosomal anchoring and scaffold construction [27]. The initial HiFi-based assembly displayed excellent contiguity. Illumina read-mapping results showed strong consistency between assembled sequences and raw reads, indicating high base-level accuracy of the assembly [23]. The final assembled genome size was slightly larger than the k-mer-estimated genome size. With Hi-C-assisted scaffolding and manual curation, the vast majority of contigs were anchored to 12 pseudochromosomes with a high anchoring rate. The Hi-C contact heatmap exhibited uniform and credible signal distribution, confirming high accuracy of chromosomal anchoring [21] with negligible misjoins and chimeric scaffolds. BUSCO evaluation revealed high gene completeness of the assembly, which fully met the requirements for subsequent gene annotation and functional analyses.

Based on the high-quality chromosome-level genome, structural annotations were performed for repetitive sequences, non-coding RNAs, and protein-coding genes of *S. carolinense*. Gene set completeness evaluation and multi-database functional annotation were also conducted [23]. Repetitive sequence annotation revealed that LTR retrotransposons constituted the largest genomic fraction, followed by DNA transposons and LINE elements, whereas SINE elements occupied only a small proportion. This structural pattern indicated prominent retrotransposon enrichment, particularly of Gypsy elements, in the *S. carolinense* genome. Non-coding RNAs occupied a low genomic proportion but represented essential components of non-coding genomic regions. BUSCO assessment yielded a gene set completeness of 94.9%, with low proportions of fragmented and missing single-copy orthologs, confirming the high reliability and quality of the predicted gene models.

GO analysis revealed that the annotated genes were enriched in terms associated with basic cellular metabolism, regulation of gene expression, and defense against biotic stresses. These genes may serve as key regulators governing the growth, development, and environmental adaptability [36] of *S. carolinense*. KEGG analysis further indicated that these genes are widely involved in basal metabolic processes and may support global metabolic network reprogramming in response to specific environmental cues [36]. KOG classification showed that most genes were annotated to core cellular processes, material metabolism, and energy balance. Meanwhile, a considerable proportion of genes in the *S. carolinense* genome remained functionally uncharacterized, providing valuable directions for future functional genomic studies. NR homology annotation revealed that *S. carolinense* shares high sequence similarity with congeneric species, including *S. dulcamara* and *S. tuberosum*. Collectively, integrated functional annotation and phylogenetic relatedness analyses can be applied to explore interspecific differences in environmental adaptability and secondary metabolism among these closely related *Solanum* species.

By generating a high-quality chromosome-level genome assembly, this study provided a critical resource for elucidating genomic structural characteristics, gene family composition, and evolutionary patterns underlying these traits [23]. Future research could now focus on identifying key genes regulating rhizome growth and development, root initiation, elongation, stress acclimation, and colonization. Such insights would clarify the molecular mechanisms governing seed germination, root growth, plant establishment, and invasive spread, paving the way for targeted genetic interventions, novel control technologies, and precise management strategies [37]. Building on this reference genome, priority should be given to developing species-specific molecular markers. These tools would enable rapid, accurate detection of seeds and root fragments at ports of entry, grain processing facilities, and nurseries, without requiring taxonomic expertise or intact plant material.

Despite these advances, current genomic understanding of *S. carolinense* was constrained by limited sampling. Future studies should address this by (1) expanding phylogeographic sampling across native and invasive ranges through whole-genome resequencing to reconstruct invasion routes and global population structure [37]; (2) integrating transcriptomic and metabolomic datasets to dissect regulatory networks underlying invasiveness and stress resistance; and (3) translating genomic findings into practical applications, such as standardized quarantine detection kits based on population-specific markers integrated with field monitoring. Ultimately, a multidisciplinary framework combining molecular biology, ecology, and quarantine science would be essential for developing a comprehensive, effective management system for this globally invasive weed.

## 4. Materials and Methods

### 4.1. Sample Preparation

Fruits of *S. carolinense* were inspected from soybean imports originating from the United States in 2024. Seeds were germinated in Petri dishes, then transplanted into nutrient soil, and grown in a controlled plant growth chamber for 50 d under a 12 h (35 °C)/12 h dark (25 °C) cycle. Root, stem, and leaf tissues were harvested from a single individual for nucleic acid extraction.

### 4.2. DNA Extraction

A total of 0.1 g of plant tissue was transferred into a sterile 2 mL grinding tube together with steel beads (3 mm in diameter), and the sample was ground at 40 Hz for 30 s. Then, 1.2 mL CTAB lysis buffer and 3 μL RNase were added to the tube; the mixture was thoroughly vortexed and incubated at 50 °C for 1 h, with gentle inversion every 20 min. After cooling to room temperature, the lysate was centrifuged at 10,000× *g* for 5 min, and the supernatant was retained. Two sequential liquid–liquid extractions were subsequently performed using an equal volume of phenol/chloroform/isoamyl alcohol (25:24:1) and chloroform/isoamyl alcohol (24:1), respectively. The supernatant was collected after each 10 min centrifugation at 10,000× *g*. The final supernatant was mixed with 0.8 volumes of pre-cooled isopropanol to precipitate genomic DNA. DNA pellets were harvested via centrifugation and washed twice with 1 mL of 75% ethanol. The pellets were air-dried to completely remove residual ethanol, and DNA was dissolved in 60 μL EB buffer at 37 °C for 30 min. The concentration and integrity of extracted DNA were determined using a NanoDrop One spectrophotometer (Thermo Fisher Scientific, Waltham, MA, USA), a Qubit 3.0 fluorometer (Thermo Fisher Scientific), and agarose gel electrophoresis (Thermo Fisher Scientific).

The qualified genomic DNA isolated from leaf tissue was submitted for subsequent PacBio HiFi, Illumina, and Hi-C sequencing, all performed by Wuhan Benagen Technology Co., Ltd., Wuhan, China.

### 4.3. Sequencing Library Construction

#### 4.3.1. PacBio HiFi SMRTbell Library Construction

Qualified high molecular weight DNA was sheared to a target size of 15 kb using the Megaruptor 3 system (Diagenode, Seraing, Belgium). Fragmented DNA was subjected to damage repair, end polishing, and A-tailing, after which ligation with SMRTbell adapters (PacBio, Menlo Park, CA, USA) was conducted. The ligation products were purified using 1× SMRTbell cleanup beads and digested with nuclease to remove linear DNA fragments. Fragments of the target size were screened with a Pippin HT system, and the quality of constructed libraries was assessed with the Agilent Fragment Analyzer System (Agilent, Santa Clara, CA, USA). Qualified DNA libraries were sequenced on the PacBio Revio platform (PacBio).

#### 4.3.2. Illumina TruSeq Short DNA Library Construction

One microgram of qualified genomic DNA was fragmented via sonication. The fragmented DNA was subjected to end repair, A-tailing, and ligation with Illumina adapters (Illumina, San Diego, CA, USA). Gel-based fragment size selection was carried out, followed by PCR enrichment for 10–12 cycles. The constructed libraries were preliminarily quantified using the Qubit 3.0 fluorometer, insert fragment sizes were assessed with the Agilent 2100 Bioanalyzer (Agilent, Santa Clara, CA, USA), and the precise library concentration was determined by qPCR. Qualified libraries were loaded onto the NovaSeq X Plus platform (Thermo Fisher Scientific) for PE150 paired-end sequencing.

#### 4.3.3. Hi-C Library Construction

Following DNA extraction, chromatin was solubilized and neutralized before overnight digestion with the MboI restriction enzyme. Biotin-labeled DNA termini were ligated within cross-linked chromatin; the DNA was subsequently reverse-crosslinked and purified. Unligated terminal biotin moieties were eliminated, and the purified DNA was sheared into fragments ranging from 200 to 600 bp, followed by end repair. Biotin-tagged DNA fragments were enriched using streptavidin magnetic beads, subjected to A-tailing, and ligated to Illumina sequencing adapters. After 12–14 cycles of PCR amplification, the constructed libraries were sequenced on the Illumina HiSeq platform to generate 2 × 150 bp paired-end reads.

### 4.4. Quality Control

Raw PacBio HiFi data were filtered by discarding polymerase reads shorter than 50 bp, reads with a quality score below 0.8, and sequences carrying adapter contamination or self-ligation artifacts (SMRT Link, v13.0; https://www.pacb.com/smrt-link/, accessed on 1 December 2025). The retained subreads were converted into HiFi circular consensus reads via SMRT Link (v13.0).

Raw Illumina sequencing reads were trimmed and filtered with fastp (v0.21.0) [38] and read quality was evaluated using FastQC (v0.11.9) [39]. Reads harboring over 5% N bases, more than 50% low-quality bases, adapter sequences or PCR duplicates were excluded [40] to generate high-quality clean reads.

Raw Hi-C sequencing data were initially processed with fastp to eliminate adapters and low-quality reads. Valid filtered reads were mapped to the draft reference genome using HiCUP. Non-uniquely aligned read pairs, invalid read fragments, and PCR duplicates were subsequently removed to yield high-confidence Hi-C contact data.

### 4.5. Genome Characterization and Assembly

Genome assembly was performed on high-quality HiFi reads using hifiasm (v0.19.8) [28]. The preliminary contig assembly was further processed with Purge_dups [41] to remove heterozygous duplications, yielding the final draft genome. Genome characteristics were estimated using k-mer analysis. Filtered reads were decomposed into k-mers and their frequency-depth distributions were obtained using Jellyfish (v2.2.1) [42]. Genome size, heterozygosity, and repeat content were estimated with GenomeScope (v2.0) [43]. To assess potential contamination, the first 50,000 sequencing reads were aligned against the NT database using BLASTn (v2.11.0, https://ftp.ncbi.nlm.nih.gov/blast/executables/blast+/2.11.0/, accessed on 3 May 2025), and taxonomic classification was performed with the taxdump tool (downloaded on 6 May 2025) to identify possible exogenous sequences. Hi-C data were subsequently used for chromosome-level assembly. Clean Hi-C reads were aligned against the draft assembly and uniquely paired-end aligned reads were screened to evaluate valid and invalid data volumes. Contigs were clustered into chromosome groups using ALLHIC (v0.9.8) [44], followed by ordering and orientation within each group. Subsequently, files were converted using 3D-DNA (v201008) [45] and Juicer (v1.6) [46], followed by manual ordering and orientation via Juicebox (v1.11.08) [47]. Redundant contigs were removed, and assembly quality was evaluated in terms of continuity, consistency, and completeness. BUSCO (v5.7.0) [29] with the embryophyta_odb10 database was utilized to assess genome assembly completeness.

### 4.6. Genome Annotation and Visualization

#### 4.6.1. Repeat Sequences and Non-Coding RNA Annotation

A de novo repeat sequence library was constructed using RepeatModeler (v2.0.6) [48], LTR_FINDER [49], and LTRharvest (v1.6.5) [50], followed by redundancy removal with LTR_retriever (v3.0.1) [51] and library establishment via teclass (v2.1.3) [52]. The resulting library was merged with RepBase (https://www.girinst.org/server/RepBase/index.php, accessed on 5 May 2025), and repeat annotation was performed using RepeatMasker (v4.1.7) and associated tools to generate a final combined transposable element (TE) dataset. Transfer RNA (tRNA) genes were identified using tRNAscan-SE (v2.0.12) [53], while ribosomal RNA (rRNA) genes were predicted with RNAmmer (v1.2) [54]. Other non-coding RNAs (ncRNAs) were identified using INFERNAL (v1.1.4) [55] based on the Rfam database [56].

#### 4.6.2. Protein-Coding Gene Annotation

Gene structure was predicted using a combination of transcriptome-based, homology-based, and ab initio methods. Next-generation and third-generation transcriptomes were filtered, aligned, and assembled to reconstruct transcripts. Protein sequences from closely related species were used for homology-based prediction with miniprot (v0.13) [57], and ab initio prediction was conducted using Augustus (v3.5.0) [58], Genscan (v1.0) [59], and glimmerhmm (v3.0.4) [60]. The final gene set was generated by integrating all data using Maker (v3.0103) [61]. Gene functions and associated pathways were further assigned based on GO [30] and KEGG databases with kofam [31]. Functional annotation of predicted proteins was performed by alignment against the NCBI NR database [32], UniProt [33], KOG, and Pfam [35] databases using Diamond (v2.1.8) [62]. InterProScan (v5.55-88.0) [34] was used to identify conserved protein sequences, motifs, and domains for gene function annotation. The completeness of genome annotation was assessed using BUSCO (v5.7.0) [29] and the embryophyta_odb10 database.

#### 4.6.3. Visualization Analysis

Chromosome-level Hi-C interaction heatmaps were generated using HiCExplorer (v3.7.5) [63] to visualize genomic spatial interaction characteristics based on contig interaction intensity and position. Protein sequences were self-aligned using BLASTp (v2.11.0), and collinear blocks were identified with MCScanX (v0.8) [64]. Finally, a circos plot was generated using the R package circlize (v0.4.16) [65] to integrate and visualize genome structure and collinearity. The bioinformatics software, databases, and parameters applied in this study are summarized in Table A1.

## 5. Conclusions

We constructed a high-quality, chromosome-scale genome assembly of *S*. *carolinense* by integrating PacBio HiFi long reads, Illumina short reads, and Hi-C scaffolding data. The assembled genome exhibits excellent contiguity and a high chromosomal anchoring rate, maintaining high genomic integrity despite its high repetitive sequence content and moderate-to-high heterozygosity. Completeness assessment confirmed the high reliability of the genome assembly. Furthermore, most predicted protein-coding genes were successfully functionally annotated, providing a robust and comprehensive genomic resource for subsequent functional gene exploration and in-depth genomic research on *S. carolinense*.

## Figures and Tables

**Table 1:** Statistics of sequencing data for *S. carolinense*.

Data Type	Total Clean Reads	Total Sequencing Volume (Gb)	Average Read Length (bp)
HiFi	5,352,783	99.89	18,660.8
Next-generation	2,655,695,670	396.96	150
Hi-C reads	589,375,206	88.40	—

**Table 2:** Statistics of *S. carolinense* genome assembly.

Item	HiFi Assembly	Hi-C Assembly
Total length (bp)	915,399,764	915,404,164
Total ungapped length (bp)	915,399,212	915,399,212
Number of sequences	128 (contigs)	12 (pseudochromosomes)
GC content (%)	35.607	35.61
N50 (bp)	51,062,083	73,173,990
N90 (bp)	8,303,358	51,062,083
Minimum sequence length (bp)	20,244	20,244
Maximum sequence length (bp)	75,629,554	120,342,759
Average sequence length (bp)	7,151,560.66	8,801,963.12
Median sequence length (bp)	58,531.5	56,066.50

**Table 3:** BUSCO evaluation statistics of *S. carolinense*.

	Assembly		Annotation	
	Proteins	Percentage (%)	Proteins	Percentage (%)
Complete BUSCOs	1530	94.8	1531	94.9
Complete Single-Copy BUSCOs	1431	88.7	1417	87.8
Complete Duplicated BUSCOs	99	6.1	114	7.1
Fragmented BUSCOs	7	0.4	8	0.5
Missing BUSCOs	77	4.8	75	4.6
Total BUSCO groups searched	1614	100.0	1614	100.0

**Table 4:** Basic statistics of dispersed repeat sequences in the *S. carolinense* genome.

Type	TE Proteins		De Novo + Repbase		Combined TEs	
	Length (bp)	% in Genome	Length (bp)	% in Genome	Length (bp)	% in Genome
DNA	1,811,710	0.20	100,481,435	10.98	100,521,263	10.98
LINE	15,303,954	1.67	69,198,810	7.56	69,961,278	7.64
SINE	0	0.00	8,089,128	0.88	8,089,128	0.88
LTR	148,227,168	16.19	485,806,018	53.07	495,747,371	54.16
LTR-Gypsy	114,318,519	12.49	222,511,562	24.31	232,561,239	25.41
LTR-Copia	28,768,230	3.14	41,618,884	4.55	47,093,658	5.14
Other	0	0.00	4271	0.00	4271	0.00
Unknown	0	0.00	39,009,728	4.26	39,009,728	4.26
Total	165,333,336	18.06	663,615,805	72.49	674,122,462	73.64

**Table 5:** Statistics of non-coding RNA annotation results in *S. carolinense*.

Type		Copy	AL (bp)	TL (bp)	% of Genome
miRNA		777	94	73,098	0.008
tRNA		1159	75	87,390	0.0095
rRNA	rRNA	15,127	128	1,931,473	0.211
	18S	25	1759	43,980	0.0048
	28S	20	4829	96,578	0.0106
	5.8S	98	120	11,787	0.0013
	5S	14,984	119	1,779,128	0.1944
snRNA	snRNA	807	117	94,620	0.0103
	CD-box	481	108	51,940	0.0057
	HACA-box	63	119	7496	0.0008
	splicing	263	134	35,184	0.0038
	scaRNA	0	0	0	0

Notes. AL, Average length; TL, Total length; bp, base pair.

**Table 6:** Protein-coding gene prediction results of the *S. carolinense* genome.

Method	Software	Species	GN	AGL (bp)	ACL (bp)	AEPG	AEL (bp)	AIL (bp)
Ab initio	GlimmerHMM		47,188	15,053.88	789.56	3.28	241.08	6269.72
	AUGUSTUS		31,562	4299.23	1156.3	4.8	240.66	826.06
Homology	Miniprot	*Solanum lycopersicum*	35,233	4098.84	1024.73	4.27	239.98	940.1
	Miniprot	*Solanum pinnatisectum*	42,512	4084.14	1091.4	3.93	277.48	1020.28
	Miniprot	*Solanum stoloniferum*	69,669	5382.92	1320.57	5.52	239.28	898.96
RNA-seq	TransDecoder		22,010	6318.52	1171.75	5.76	310.77	952.49
Integration	MAKER		26,844	6851.66	1166.87	5.28	292.24	1240.47
Final set	Anno-self		32,206	5209.52	1149.76	4.78	312.49	982.1

Note. GN, Gene number; AGL, Average gene length; ACL, Average CDS length; AEPG, Average exon per gene; AEL, Average exon length; AIL, Average intron length; bp, base pair. TransDecoder (v5.7.0) was run with default parameters.

**Table 7:** Functional annotation results of predicted protein-coding genes.

Database	Count	Percentage
GO	18,552	57.60%
KEGG	20,058	62.28%
Pathway	10,317	32.03%
KOG	3522	10.94%
NR	30,477	94.63%
UniProt	30,496	94.69%
InterPro	30,508	94.73%
Pfam	22,731	70.58%

## Data Availability

The PacBio HiFi reads and Hi-C reads generated in this study have been deposited in the Genome Sequence Archive (GSA) [66] of the National Genomics Data Center (NGDC) [67], Beijing Institute of Genomics, Chinese Academy of Sciences/China National Center for Bioinformation, under accession number CRA040133 (https://ngdc.cncb.ac.cn/gsa, accessed on 1 April 2026). The chromosome-level genome assembly of *S. carolinense* is available on Zenodo with the DOI 10.5281/zenodo.19572094.

## Appendix A

**Table A1:** Summary of software and database versions and parameters.

Software/Databases	Version	Parameters
Hifiasm [28]	0.19.8	default
BUSCO [29]	5.7.0	-m prot -c 16
GO [30]	2024-05-29	-
Kofam [31]	2024-05-27	-
NR [32]	2024-02-07	-
UniProt [33]	2024-05-29	-
InterProScan [34]	5.55-88.0	default
Pfam [35]	37.0	-
Fastp [38]	0.21.0	-detect_adapter_fo r_pe
FastQC [39]	0.11.9	-o.
purge_dups [41]	1.2.5	-2
Jellyfish [42]	2.2.1	count -C -m 19 -s 1 G -g generators -G 4/stats
GenomeScope [43]	2.0	-i histo_all -k kmer _size -o./genomes cope -p ploidy
BLASTn	2.11.0	-evalue 1e-5 -max_target_seqs 1 -outfmt ‘6 qaccver saccver pident length mismatch gapopen qstart qend sstart send evalue bitscore sscinames sskingdoms stitle’
ALLHIC [44]	0.9.8	-e GATC
3D-DNA [45]	201008	q 30
Juicer [46]	1.6	-g material -s MboI -t 30 -S early
Juicebox [47]	1.11.08	Coverage
RepeatModeler [48]	2.0.6	-database mydb -threads 16
LTR_FINDER [49]	Official release of LTR_FINDER_parallel	-threads 16 - harvest_out -size 1000000 -time 300
LTRharvest [50]	1.6.5	-minlenltr 100 - maxlenltr 7000 - mintsd 4 -maxtsd 6 - motif TGCA - motifmis 1 -similar 85 -vic 10 -seed 20 - seqids yes
LTR_retriever [51]	3.0.1	-threads 16 -noanno
Teclass [52]	2.1.3	default
RepeatMasker	4.1.7	-noLowSimple - pvalue 0.0001
tRNAscan-SE [53]	2.0.12	-E -j tRNA.gff -o tRNA.result -f tRNA.struct –thread 16
RNAmmer [54]	1.2	-S euk -m tsu,lsu,ssu
INFERNAL [55]	1.1.4	–cut_ga –rfam –nohmmonly –fmt 2
Rfam [56]	14.10	-
Miniprot [57]	0.13	–gff -Iut50
AUGUSTUS [58]	3.5.0	–uniqueGeneId=true –noInFrameStop=true –gff3=on –strand=both
Genscan [59]	1.0	default
GlimmerHMM [60]	3.0.4	-f -g
MAKER [61]	3.01.03	default
Diamond [62]	2.1.8	–evalue 1e-05
HiCExplorer [63]	3.7.5	-dpi 300 -log1p -colorMap Reds -clearMaskedBins -rotationX 45 -vMin 11
MCScanX [64]	0.8	default
Circlize [65]	0.4.16	default
